# Performance of ChatGPT vs. HuggingChat on OB-GYN Topics

**DOI:** 10.7759/cureus.56187

**Published:** 2024-03-14

**Authors:** Gabrielle Kirshteyn, Roei Golan, Mark Chaet

**Affiliations:** 1 Obstetrics and Gynecology, Florida State University College of Medicine, Tallahassee, USA; 2 Urology, Florida State University College of Medicine, Tallahassee, USA; 3 Pediatric Surgery, Florida State University College of Medicine, Orlando, USA

**Keywords:** chatgpt 3.5, medical student assessment, nbme examinations, gynecology and obstetrics, artificial intelligence in medicine

## Abstract

Background

While large language models show potential as beneficial tools in medicine, their reliability, especially in the realm of obstetrics and gynecology (OB-GYN), is not fully comprehended. This study seeks to measure and contrast the performance of ChatGPT and HuggingChat in addressing OB-GYN-related medical examination questions, offering insights into their effectiveness in this specialized field.

Methods

ChatGPT and HuggingChat were subjected to two standardized multiple-choice question banks: Test 1, developed by the National Board of Medical Examiners (NBME), and Test 2, gathered from the Association of Professors of Gynecology & Obstetrics (APGO) Web-Based Interactive Self-Evaluation (uWISE). Responses were analyzed and compared for correctness.

Results

The two-proportion z-test revealed no statistically significant difference in performance between ChatGPT and HuggingChat on both medical examinations. For Test 1, ChatGPT scored 90%, while HuggingChat scored 85% (p = 0.6). For Test 2, ChatGPT correctly answered 70% of questions, while HuggingChat correctly answered 62% of questions (p = 0.4).

Conclusion

Awareness of the strengths and weaknesses of artificial intelligence allows for the proper and effective use of its knowledge. Our findings indicate that there is no statistically significant difference in performance between ChatGPT and HuggingChat in addressing medical inquiries. Nonetheless, both platforms demonstrate considerable promise for applications within the medical domain.

## Introduction

Large language models (LLMs), a subset of artificial intelligence (AI), are constructed of vast computational algorithms that analyze data and train the machine to make autonomous conclusions [1]. Such conclusions can be used to answer user-prompted questions and demands [2]. ChatGPT is included in this category of LLMs and has experienced significant growth in user engagement since its introduction in November 2022 [3]. Unlike ChatGPT, which operates on a proprietary framework, Hugging Face’s chatbot, HuggingChat, is developed on an open-source platform, allowing for community-based contributions and enhancements [4,5].

These programs are rapidly evolving, and their knowledge could be used as a resource in many fields [6]. However, the consensus to use LLMs as a tool in medicine has not yet been achieved, as the reliability of the information they provide is not yet fully understood [6-8]. As such, previous studies of AI performance in medical examinations have found varying and contradicting results [9-11]. While studies have examined ChatGPT’s capabilities in undertaking medical exams [12], there is a notable lack of comparisons between its scores and those of other AI entities, like HuggingChat [10].

Analyzing the knowledge of AI through its responses to medical questioning could elucidate the strengths of using its resources in the medical field. There is a paucity of information on AI’s knowledge of obstetrics and gynecology (OB-GYN) topics, especially in terms of which platform is more equipped with information. This paper aimed to quantify and compare the performance of two AI modalities, ChatGPT and HuggingChat, on medical examination questions testing OB-GYN proficiency.

## Materials and methods

Examination data set

In evaluating the performance of the two AI modalities, ChatGPT and HuggingChat, each was subjected to two standardized question banks: Test 1 and Test 2. Test 1 was composed of 20 questions and was compiled from the Obstetrics & Gynecology Sample Items developed by the National Board of Medical Examiners (NBME). This test bank is comprised of 20 questions and is available for free online on the company’s website. Test 2 was composed of 50 questions gathered from the Comprehensive 1: 50 question exam (2022) created by the Association of Professors of Gynecology & Obstetrics (APGO) Web-Based Interactive Self-Evaluation (uWISE). APGO’s uWISE is an American College of Obstetricians and Gynecologists (ACOG)-endorsed interactive self-assessment tool designed to prepare medical students for the OB-GYN subject examination. We used each question bank in its entirety as it is found online. The passing score for each exam is 70%, which was determined by each of the test makers (NBME and APGO).

The 70 questions included in the examination data set only included textual prompts and did not include images. All questions were multiple-choice formatted in that the question prompt was followed by its associated set of multiple-choice answers.

In the third and fourth years of medical school, students are given subject “Shelf” examinations to test their proficiency in various specialties, including internal medicine, surgery, and OB-GYN. These examinations are created and administered by the NBME. For the preparation of the OB-GYN exam, ACOG recommends and endorses practice questions made by APGO’s uWISE.

Data analysis

Data collection was performed on September 18, 2023. The ChatGPT (GPT-3.5) August 3, 2023 version was used. ChatGPT operates on a proprietary framework [4]. The HuggingChat model meta-llama/Llama-2-70b-chat-hf was used. HuggingChat is developed on an open-source platform, allowing for community-based contributions and enhancements [5]. We manually entered questions into the ChatGPT and HuggingChat chat prompts. We then manually recorded the AI’s multiple-choice answer and directly copied its explanation into a spreadsheet.

To ascertain the performance distinction between ChatGPT and HuggingChat on two separate medical examinations, we employed a two-proportion z-test for each examination. The first medical examination (Test 1) consisted of 20 questions, while the second examination (Test 2) comprised 50 questions. The number of correct responses by each AI system on each test was recorded. The significance level was set at α = 0.05 for determining statistical significance.

## Results

The two-proportion z-test revealed no statistically significant difference in performance between ChatGPT and HuggingChat on both medical examinations. For Test 1, ChatGPT correctly answered 18 out of 20 questions (90%), while HuggingChat correctly answered 17 out of 20 questions (85%) (p = 0.6). For Test 2, ChatGPT correctly answered 35 out of 50 questions (70%), in contrast to HuggingChat's 31 correct answers out of 50 questions (62%) (p = 0.4) (Table 1). On Test 1, a wrong answer was commonly generated by both AI modalities on one question. On Test 2, there were seven questions that both HuggingChat and ChatGPT got incorrect.

**Table 1:** Performance of ChatGPT and HuggingChat on Test 1 and Test 2

AI system	Test	Questions answered correctly	Performance (%)	Outcome	p-value
ChatGPT	Test 1	18/20	90%	Pass	0.6
HuggingChat	Test 1	1720	85%	Pass	0.6
ChatGPT	Test 2	35/50	70%	Pass	0.4
HuggingChat	Test 2	31/50	62%	Fail	0.4

## Discussion

Determining the reliability of AI’s information database can help justify its use as a resource in the medical field. In this paper, we analyzed the responses provided by AI programming to user-prompted questioning of OB-GYN topics. We found that the two LLMs can compute medical questioning and formulate responses. Although ChatGPT outperformed HuggingChat on both examinations, the differences in their performances were not statistically significant.

ChatGPT received a passing score on Test 1 (NBME), displaying adequate knowledge of OB-GYN topics. Prior research by Mackey et al. had similar results, as they found ChatGPT to score 94% on the OB-GYN topics of a question bank written by AMBOSS [13]. Riedel et al. also used OB-GYN questions from AMBOSS as well as OB-GYN course exams at the University Hospital of the Technical University of Munich to prompt ChatGPT [14]. Although they prompted their questions in the German language, ChatGPT was able to pass both examinations [14]. However, contradictory results were found by Cohen et al., as they calculated a score of 38.7% correct, a failing score, on the 150-question Israeli medical examination for OB-GYN residents [11]. This discrepancy could possibly be explained by the machine’s inability to compute in Hebrew, as they prompted their questions in that language.

ChatGPT received a passing score of 70% on APGO’s uWISE, which is consistent with the results Koch et al. gathered. Their project included the same version of ChatGPT (3.5), and their question prompts were also created by APGO. ChatGPT was found to score 73.5% and 71.4%, respectively, on two different attempts [15].

There is a lack of literature on HuggingChat’s performance on examinations in the medical field. From our experience, HuggingChat had adequate information to answer questions written by the NBME, which was evident by its performance on Test 1 (85%). However, the program was unable to correctly answer enough of APGO’s questions to pass (62%). This could be due to a difference in computational algorithms, a smaller information database, a greater number of questions posed, or question characteristics.

There were a handful of questions where both AI programs produced an incorrect answer. On Test 1, there was one question, while on Test 2, there were seven. Surprisingly, when analyzing the responses provided by the programs, there was only one question out of eight where both programs stated the same incorrect answer. This question was found on Test 2 and described a 17-year-old female athlete who recently developed acne, facial hair growth, menstrual irregularity, and a nodular lesion on the left buttock. The question asked was to choose the next step in management. While the correct answer, determined by APGO, was to test for anabolic steroid use, both AI modalities chose to test her 17-hydroxyprogesterone level. ChatGPT chose against the correct answer with the reasoning that “A urine anabolic steroid test is not typically used as a first-line diagnostic test for assessing virilization. It is more relevant in the context of suspected illicit steroid use,” while HuggingChat stated, “A urine anabolic steroid test is unlikely to be helpful in this case, as there is no evidence to suggest that the patient is using anabolic steroids.” It seems that the AI algorithm picked up on the hirsutism but ignored the mass on the buttock, which could hint toward the use of injections. This leads us to believe that the programs struggle to make assumptions and read between the lines, as they did not analyze aspects that were not explicitly stated. There was no noticeable pattern for the other seven questions. The missed topics included ethics, calculating an Apgar score, preeclampsia, preterm deliveries, and fetal demise. Half of the eight questions asked, “What is the next step in management?”.

The medical expertise of LLMs extends beyond the realm of OB-GYN. ChatGPT has demonstrated proficiency in successfully navigating questions from the United States Medical Licensing Examination (USMLE) Steps 1, 2, and 3 [16]. However, it is noteworthy that the model encountered challenges in examinations related to radiology, with a performance rate of 69%, and urology, where success rates were 42.3% and 30% [12,17].

Although the LLMs’ response generation has not yet been perfected for accuracy, there are still numerous advantages to their use in the medical field. For educational purposes, it provides accessible, detailed information tailored to individualized topics. It also provides a possibility for conversation (with the AI) about the subject, which could enhance comprehension and fill knowledge gaps. As for the clinical aspect of medicine, AI can serve as a valuable asset in swiftly providing information, formulating differential diagnoses, and suggesting appropriate treatment options.

Limitations

There are several limitations to this study. The reproducibility is limited as the AI platforms are consistently evolving and gaining more knowledge. With the increased number of searches and data input, the machines are adapting new expertise and improving responses. Therefore, the versions we used in this study may not represent the updated versions present at the time of publication. In addition, the programs are only as knowledgeable as the data they are programmed with, which serves as an additional limitation. We were limited to the use of text-only questions as the programs were unable to decipher photographs. Lastly, our searches were also written in English, so our results cannot be generalized to include their performances in other languages.

## Conclusions

Awareness of the strengths and weaknesses of AI allows for the proper and most effective use of them. Our data provides valuable insight into the knowledge and accuracy of ChatGPT and HuggingChat on OB-GYN topics. ChatGPT was able to attain a passing score on both examinations, whereas HuggingChat’s lack of medical knowledge led to a failure of Test 2. The programs serve to provide relevant information on a particular subject, but the results should be verified before their acceptance, as some may be incorrect. Although both platforms have room for improvement, the tools have promising potential.
